# Efficacy of a mobile health application on self-management among Japanese patients with chronic kidney disease

**DOI:** 10.1007/s10157-025-02713-9

**Published:** 2025-06-02

**Authors:** Reina Suetsugu-Ishizawa, Hirofumi Sakuma, Motoki Matsuki, Seiji Itano, Hajime Nagasu, Hiroshi Morinaga, Haruhito A. Uchida, Takashige Kuwabara, Toshiyuki Imasawa, Kazuki Yamada, Naoki Nakagawa

**Affiliations:** 1 Division of Cardiology and Nephrology, Department of Internal Medicine, Asahikawa Medical University, Midorigaoka-higashi 2-1-1-1, Asahikawa, Japan; 2 Department of Nephrology and Hypertension, Kawasaki Medical School, Okayama, Japan; 3 Department of Nephrology, Rheumatology, Endocrinology and Metabolism, Okayama University Faculty of Medicine, Dentistry and Pharmaceutical Sciences, Okayama, Japan; 4 Department of Comprehensive Therapy for Chronic Kidney Disease, Okayama University Faculty of Medicine, Dentistry and Pharmaceutical Sciences, Okayama, Japan; 5 Department of Chronic Kidney Disease and Cardiovascular Disease, Okayama University Faculty of Medicine, Dentistry and Pharmaceutical Sciences, Okayama, Japan; 6 Department of Nephrology, Kumamoto University Graduate School of Medical Sciences, Kumamoto, Japan; 7 Department of Nephrology, National Hospital Organization Chiba-Higashi National Hospital, Chiba, Japan; 8 Department of Internal Medicine, Kitasaito Hospital, Hokkaido, Japan

**Keywords:** Medication adherence, Chronic kidney disease, Mobile health application

## Abstract

**Objective:**

Poor medication adherence undermines evidence-based treatment efficacy in chronic kidney disease (CKD). This study evaluated whether the mobile health (mHealth) application, *KaKalink,* improves patient self-management by delivering health-related information.

**Methods:**

In this 32-week, open-label, pre- and post-intervention trial, 119 patients (74 men, 62%) with CKD were recruited from nephrology outpatient hospitals. An 8-week non-intervention period (Period A) was followed by a 24-week intervention period (Period B). During the intervention period, the participants received health-related information via *KaKalink* every two weeks. Changes in the medication adherence input rate and blood pressure (BP) and body weight (BW) measurement input rates were evaluated. A satisfaction survey was conducted using *KaKalink*.

**Results:**

The mean (standard deviation) patient age was 53.3 (11.9) years. The mean morning and evening BP in pre-Period A were 121.0 (9.5)/76.0 (7.8) mmHg and 117.7 (11.0)/72.5 (9.2) mmHg, respectively. The mean medication adherence input rate decreased from 68.0% (95% confidence interval [CI] 60.1–75.8%) in Period A to 60.2% (95% CI 52.1–68.4%) in Period B (p < 0.0001), with women showing higher adherence than men. Similar declining trends were observed for BP and BW measurement input rates. Most participants reported high satisfaction with the mHealth application usage via the questionnaire survey.

**Conclusions:**

The information provided via *KaKalink* did not significantly improve adherence or changes in BP or BW; however, most participants perceived the application to be a useful and highly satisfactory tool for enhancing their self-management.

**Supplementary Information:**

The online version contains supplementary material available at 10.1007/s10157-025-02713-9.

## Introduction

Chronic kidney disease (CKD) is a major global public health concern [1, 2], with approximately 13 million patients in Japan, a number that has been rising [3]. The progression of CKD to end-stage renal disease increases the demand for renal replacement therapy (RRT), which reached about 350,000 cases in Japan by 2020, imposing a considerable economic burden on healthcare systems [4]. From the perspective of reducing national healthcare costs [5–7], preventing the progression of CKD, particularly through managing lifestyle-related diseases such as diabetic kidney disease (DKD) and nephrosclerosis, is considered a national priority to reduce the number of patients receiving RRT.

Despite guidelines recommending medication, dietary, and physical interventions for CKD management, nonadherence to prescribed medications remains a significant barrier, with approximately 39% of CKD patients affected [8]. Several factors contribute to non-adherence, including forgetfulness, communication barriers, socioeconomic challenges, and lack of motivation [9]. Effectively addressing non-adherence is critical to improving patient outcomes [10]. In recent years, various mobile health (mHealth) applications have emerged as effective tools for improving medication adherence by enhancing patient awareness and supporting self-management [11, 12]. *KaKalink*, an mHealth application, facilitates self-management for lifestyle-related diseases through health-related text messages and communication features, aiming to improve patient engagement and adherence.

This study aimed to examine whether providing health-related information to patients with CKD through an mHealth application called *KaKalink* improved their self-management and satisfaction with the mHealth application usage.

## Methods

### Study design

**Fig. 1:**
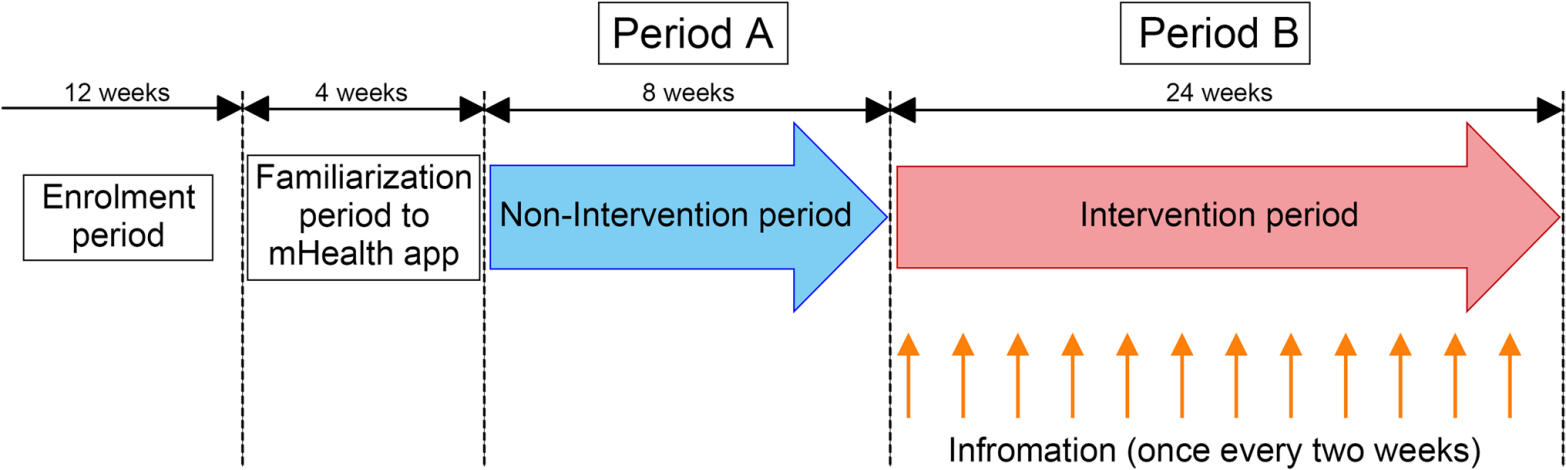
Schematic overview of the pre- and post-interventional trial

This was an open-label pre- and post-intervention trial. This study investigated whether an intervention using the mHealth application *KaKalink* improves medication adherence in patients with CKD compared with pre-intervention. The total duration was 32 weeks, with the first 8 weeks as the non-intervention period (Period A) and the last 24 weeks as the intervention period (Period B). A pre-trial period was set to allow the participants to familiarize themselves with the application following trial enrollment. Figure 1 shows the protocol schedule in this study. This study was conducted at seven hospitals (four university and three regional core hospitals) with outpatient nephrology departments. Participant enrollment began on April 7, 2023 and ended on July 14, 2023. The trial commenced on August 1, 2023 and was completed on March 11, 2024. Written informed consent was obtained from all participants. The Ethics Committee of Asahikawa Medical University approved the study design, and all participating sites approved the study (No. C22124). The study protocol is registered in the UMIN Clinical Trials Registry (UMIN000050427).

### Participants

Patients with stage G2–G4 CKD from the nephrology outpatient departments of the participating hospitals were enrolled. The patients were cared for by their nephrologists according to the Japanese Clinical Practice CKD guidelines. The inclusion criteria were (1) age ≥ 20 years, (2) CKD stage G2–G4 (estimated glomerular filtration rate 15–90 [mL/min/1.73 m^2^]), (3) use of antihypertensive medication for at least 4 weeks before enrollment, (4) outpatients, and (5) provision of written informed consent. The exclusion criteria were (1) rapidly progressive glomerulonephritis, (2) polycystic kidney disease, (3) prior renal transplantation, and (4) deemed inappropriate by a physician. A total of 121 patients from seven hospitals were registered, and two individuals did not agree to participate; therefore, the sample included 119 participants.

### Use of the KaKalink application

*KaKalink* was provided by Value-Promotion Co. Ltd (Tokyo, Japan). All participants installed the *KaKalink* application on their mobile phones after enrollment and were instructed to use the self-management features. The application provides various health-related information to participants via notifications and offers medication management and self-measured data recording features. The medication management feature involves sending alerts at scheduled times to prompt medication intake. After the patients took their medication, a button was pressed to record their intake and the time of administration. The self-measurement recording feature enabled participants to record self-measured data, including their blood pressure (BP) and body weight (BW).

### Intervention

The participants began the trial with an 8-week non-intervention period (Period A) during which the participants did not receive any messages from the app, followed by a 24-week intervention period (Period B). During Period B, participants received various health-related information every two weeks (Supplemental Table 1).

**Table 1:** Questionnaire survey for the mHealth application

Questionnaire survey for the mHealth application	
a)	Do you think the knowledge about medication and blood pressure values are increased?
b)	Do you think the consciousness of self-management is increased?
c)	Do you think the receiving regular notifications from the hospital is appreciate?
d)	Do you think the reviewing notification content at any time is useful?
e)	Do you think the medication management feature is convenient?
f)	Do you think the self-measurement recording feature is convenient?

### Endpoint

The primary endpoint was the change in the medication adherence input rate between Periods A and B. The medication adherence input rate was calculated as the number of times participants recorded medication intake via “*Kakalink*” as a percentage of the total number of prescribed medications. The secondary endpoints were changes in the following parameters: (1) BP measurement input rate, (2) BW measurement input rate, (3) BP values, and (4) health-related information reading rates. The BP measurement input rate was calculated as the proportion of measurement times relative to the total number of scheduled morning and evening measurements at home. The BW measurement (once per day) input rate was calculated similarly. These input rate endpoints as for input rate were measured as the average over the entire period with a stratified analysis by age groups. Additionally, Periods A and B were divided into two and three phases, respectively, to further investigate differences in input rate within each period. BP values are represented by the median, interquartile range, maximum, and minimum at pre-Period A (0 week), pre-Period B (8 week), and post-Period B (32 week). The BP value at each time point was the average of the three days before and after each time point. Additionally, the health-related information reading rates were calculated as the proportion of actual readings via *Kakalink* to a total of 16 instances.

### Satisfaction questionnaire survey for the mHealth application

The satisfaction questionnaire with the use of mHealth application *Kakalink* included four questions (Table 1) and was composed of five subscales: agree, somewhat agree, neutral, somewhat disagree, and strongly disagree. The application functionality questionnaire included two questions (Table 1) and comprised five subscales: strongly convenient, convenient, neutral, somewhat inconvenient, and strongly inconvenient.

### Statistical analysis

In a previous study on the medication adherence input rate of calcimimetics for Japanese patients undergoing dialysis, the rate of these drugs was 93.0% [13]. This percentage is relatively higher than that of non-dialysis patients because they receive more frequent opportunities for medical counseling from their healthcare professionals because of their thrice-weekly clinic visits. Thus, the medication adherence input rate among patients with CKD was hypothesized to be 90.0 ± 15.0%. In this situation, we estimated that a sample size of 100 participants would provide 80% power and a 5% level of significance to detect a 5% increase in the rate. This sample size allowed for a 20% loss of follow-up during the trial. The medication adherence input rate, BP measurement input rate, and BW measurement input rate are represented as mean and 95% confidence intervals (CIs) in both periods. The BP values are represented as box-and-whisker plots for pre-Period A, pre-Period B, and post-Period B. To investigate the effect of *KaKalink*, paired t-tests were used to compare the medication adherence input rates, BP measurement input rates, and BW measurement input rates between Periods A and B including a stratified analysis by age groups. BP values at each time point were also compared using one-way analysis of variance with Holm-Sidak’s multiple comparison test. Statistical significance was set at p values < 0.05. All statistical analyses were performed using GraphPad Prism 10 for Windows, Version 10.2.3 (GraphPad Software, San Diego, CA, USA).

## Results

### Participants’ characteristics

A total of 119 participants (74 men, 62%) from seven hospitals completed the 32-week trial (Table 2). The mean (standard deviation) patient age was 53.3 (11.9) years. In pre-Period A, the mean morning and evening BP were 121.0 (9.5)/76.0 (7.8) mmHg and 117.7 (11.0)/72.5 (9.2) mmHg, respectively (Table 2).

**Table 2:** Demographic clinical characteristics of the whole study population

	Mean ± SD or N (%)
Male sex	74 (62.2)
Age (years)	53.3 ± 11.9
20–29	3 (2.5)
30–39	12 (10.1)
40–49	31 (26.0)
50–59	35 (29.4)
60–69	24 (20.2)
70–79	14 (11.8)
Body weight (kg)	69.6 ± 15.8
G stage of chronic kidney disease	
G2	27 (22.7)
G3	67 (56.3)
G4	25 (21.0)
Number of daily medications	3.4 ± 3.4
by age	
20–29	3.3 ± 3.5
30–39	3.1 ± 2.7
40–49	4.1 ± 3.9
50–59	3.8 ± 3.9
60–69	2.9 ± 2.7
70–79	2.4 ± 2.1
Blood pressure at baseline (mmHg)	
Morning systolic blood pressure	121.0 ± 9.5
Morning diastolic blood pressure	76.0 ± 7.8
Evening systolic blood pressure	117.7 ± 11.0
Evening diastolic blood pressure	72.5 ± 9.2

Values are mean ± SD (standard deviation), or N (%)

### Primary endpoint

**Fig. 2:**
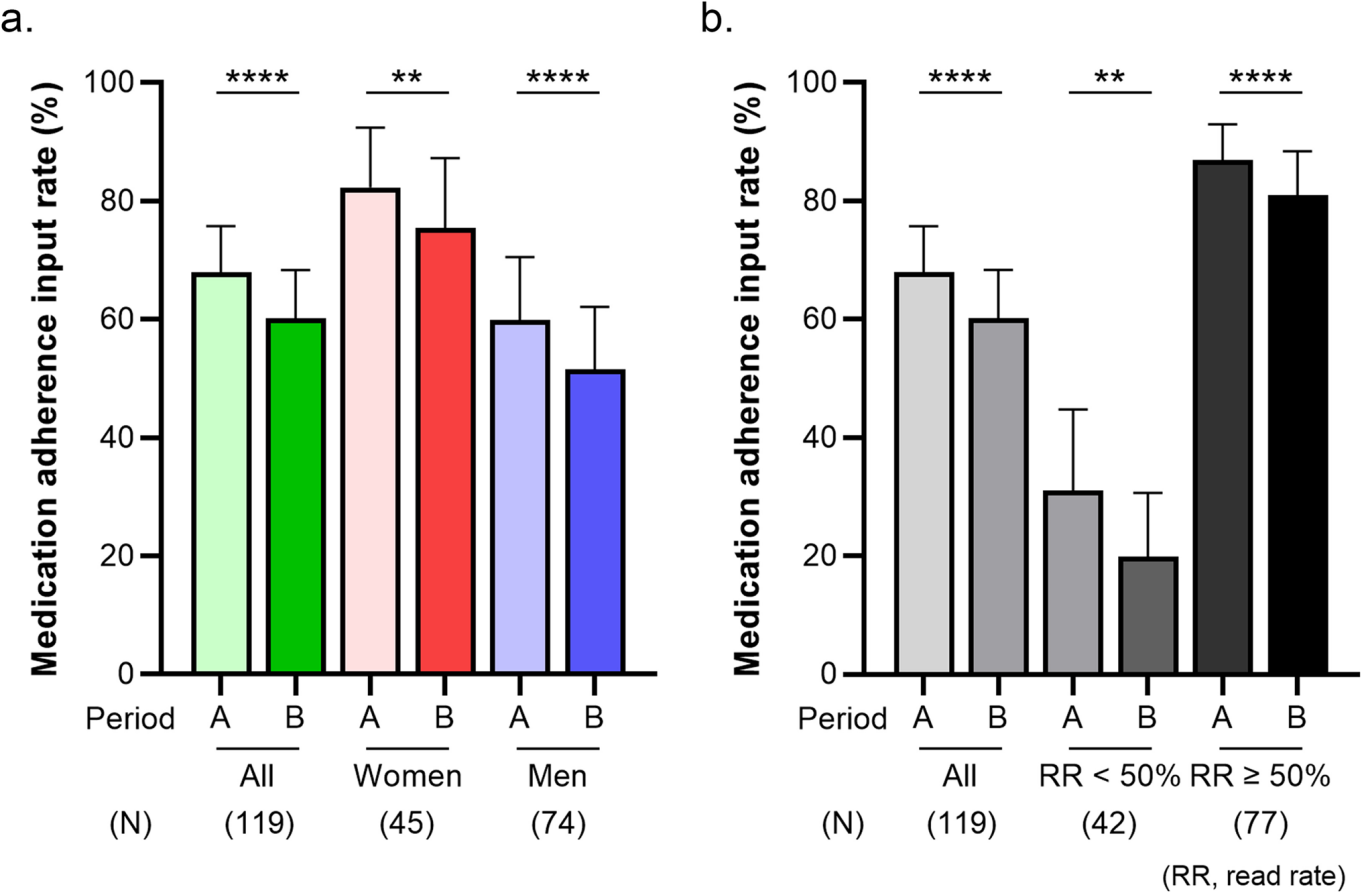
Medication adherence input rates in Period A and Period B according to sex (**a**) and information read rate (**b**). Each bar graph and error bar represent the mean and 95% confidence interval. Statistical analysis is performed using analysis of a paired t-test. **p < 0.01, ****p < 0.0001, compared with Period A

The mean medication adherence input rate for all participants declined from 68.0% (95% CI 60.1–75.8%) in Period A to 60.2% (95% CI 52.1–68.4%) in Period B (p < 0.0001, Fig. 2a). Among women, the rate also declined from 82.3% (95% CI 72.1–92.4%) in Period A to 75.4% (95% CI 63.6–87.2%) in Period B (p < 0.01, Fig. 2a). A similar trend was observed in men, with the rate declining from 59.9% (95% CI 49.2–70.5%) in Period A to 51.5% (95% CI 41.0–62.1%) in Period B (p < 0.0001, Fig. 2a). Notably, the rate in women was significantly higher than that in men during each period. By age group, the mean medication adherence input rate in the 50 s had the highest input rates, while those below the 50 s had the lowest rates (Figure S1a). In all age groups, the input rate was significantly lower in Period B than in Period A (Figure S1a). When Periods A and B were subdivided, the mean medication adherence input rates also declined over time (Figure S2a).

We further compared the rate in each period according to the information read rate in Period B. Among participants with information read rates above 50%, the rate declined significantly from 86.9% (95% CI 80.9–93.0%) in Period A to 81.0% (95% CI 73.6–88.4%) in Period B (p < 0.0001, Fig. 2b). A similar trend was observed in participants with read rates below 50%, with the rates declining from 31.0% (95% CI 17.2–44.8%) in Period A to 19.9% (95% CI 9.0–30.7%) in Period B (p < 0.01, Fig. 2b). Participants with higher information read rates demonstrated a tendency toward higher medication adherence input rates.

### Secondary endpoints

#### BP and BW measurement input rate

**Fig. 3:**
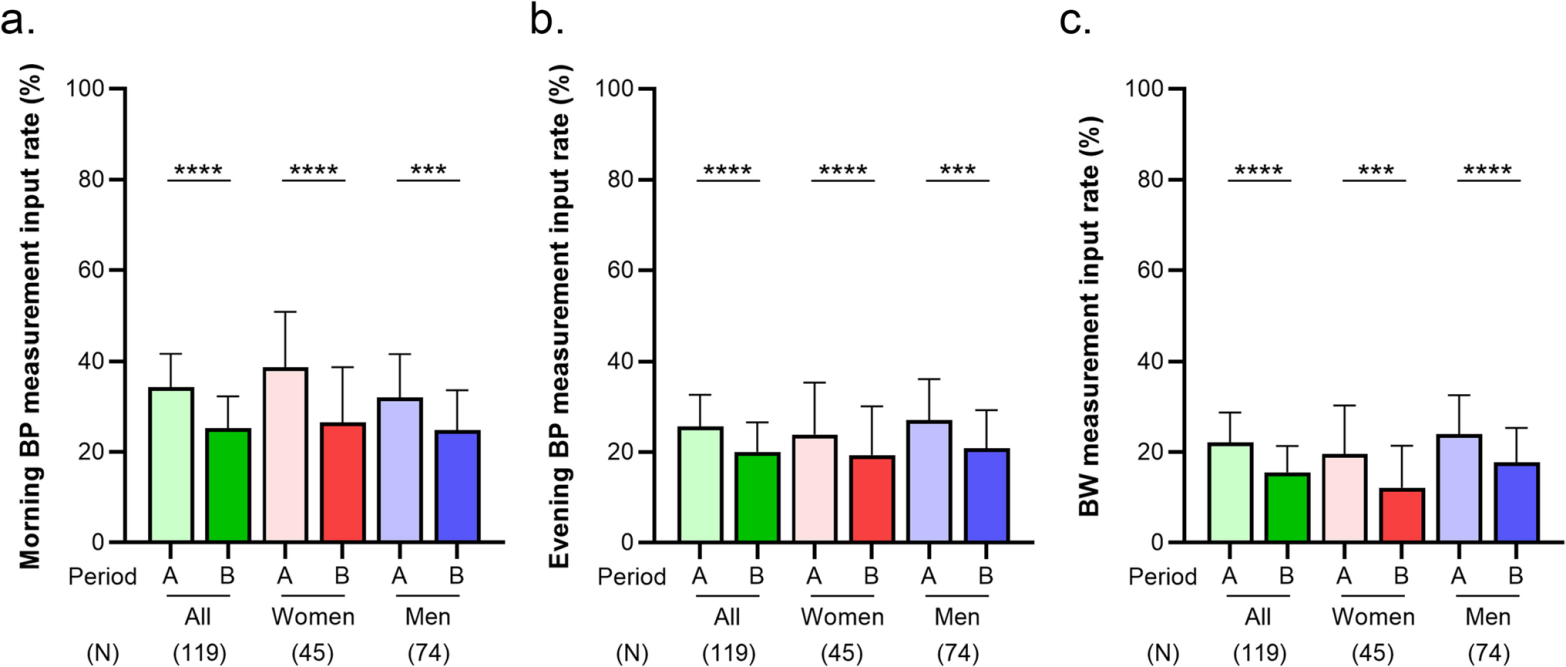
Morning blood pressure (BP) measurement input rates (**a**), evening BP measurement input rates (**b**), and body weight measurement input rates (**c**) in Period A and Period B according to sex. Each bar graph and error bar represent the mean and 95% confidence intervals. Statistical analysis is performed using analysis of a paired t-test. ***p < 0.001, ****p < 0.0001, compared with Period A

The morning BP measurement input rate for all participants declined from 34.3% (95% CI 26.9–41.6%) in Period A to 25.3% (95% CI 18.3–32.3%) in Period B (p < 0.0001, Fig. 3a). Among women, the rate declined from 38.7% (95% CI 26.6–50.9%) in Period A to 26.6% (95% CI 14.4–38.7%) in Period B (p < 0.0001, Fig. 3a). A similar trend was observed in men, with the rate declining from 32.1% (95% CI 22.6–41.6%) in Period A to 24.9% (95% CI 16.1–33.6%) in Period B (p < 0.001, Fig. 3a). Notably, the rate in women was consistently higher than that in men during each period. A similar trend was observed for the evening BP measurement input rates (Fig. 3b).

The BW measurement input rate for all participants declined from 22.1% (95% CI 15.5–28.7%) in Period A to 15.5% (95% CI 9.7–21.3%) in Period B (p < 0.0001, Fig. 3c). Among women, the rate declined from 19.6% (95% CI 8.9–30.3%) in Period A to 12.2% (95% CI 2.9–21.4%) in Period B (p < 0.001, Fig. 3c). A similar trend was observed in men, with the rate declining from 23.9% (95% CI 15.3–32.6%) in Period A to 17.7% (95% CI 10.0–25.4%) in Period B (p < 0.001, Fig. 3c). Notably, the rate in men was consistently higher than that in women during each period. By age group, the BP and BW measurement input rates in those aged > 60 years had the highest input rates, while those in below the 50 s had the lowest rates (Figure S1b–d). In all age groups, the input rate was significantly lower in Period B than in Period A (Figure S1b–d). When Periods A and B were subdivided, the mean BP and BW measurement input rates also declined over time (Figure S2b–d).

#### Changes in BP values

**Fig. 4:**
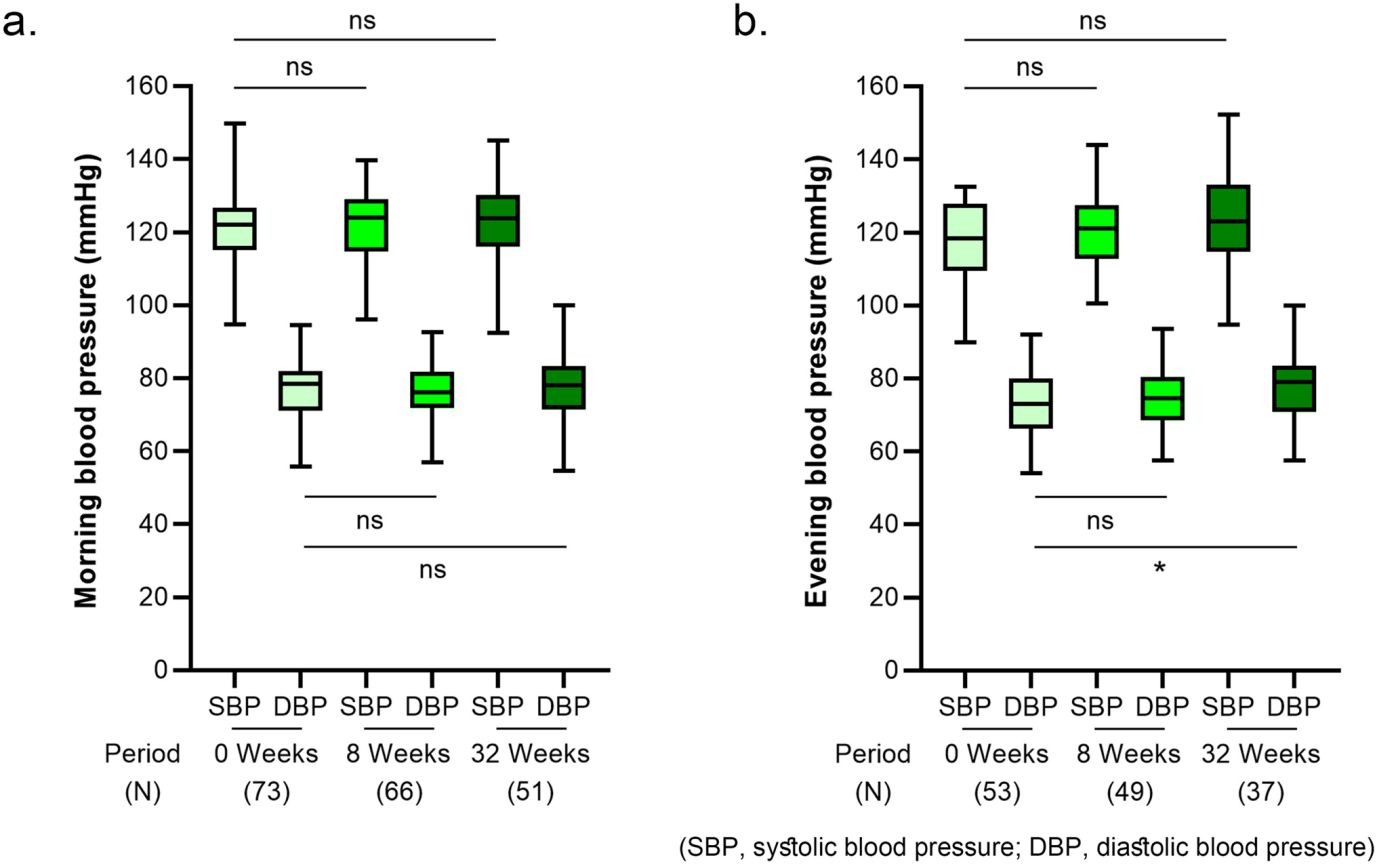
Values of morning BP (**a**) and evening BP (**b**) at the pre-Period A (0 weeks), pre-Period B (8 weeks), and post-Period B (32 weeks). Each box-and-whisker plot represents the median, interquartile range, maximum, and minimum. Statistical analysis is performed using analysis of a one-way analysis of variance with Holm-Sidak’s multiple comparison test. *p < 0.05, compared with 0-week

The morning BP values between pre-Period A (0 weeks), pre-Period B (8 weeks), and post-Period B (32 weeks) were 121.0 (9.5)/76.0 (7.8) mmHg, 121.5 (9.9)/76.0 (6.8) mmHg, and 122.4 (11.0)/77.2 (9.2) mmHg, respectively. No significant differences were observed in the morning BP values during each period (Fig. 4a). The evening BP values between pre-Period A (0 weeks), pre-Period B (8 weeks), and post-Period B (32 weeks) were 117.7 (11.0)/72.5 (9.2) mmHg, 119.9 (10.1)/74.5 (8.2) mmHg, and 123.0 (12.8)/77.3 (9.7) mmHg, respectively. Significantly higher diastolic BP values were observed post-Period B than pre-Period A (Fig. 4b).

#### Satisfaction questionnaire survey for the mHealth application

**Fig. 5:**
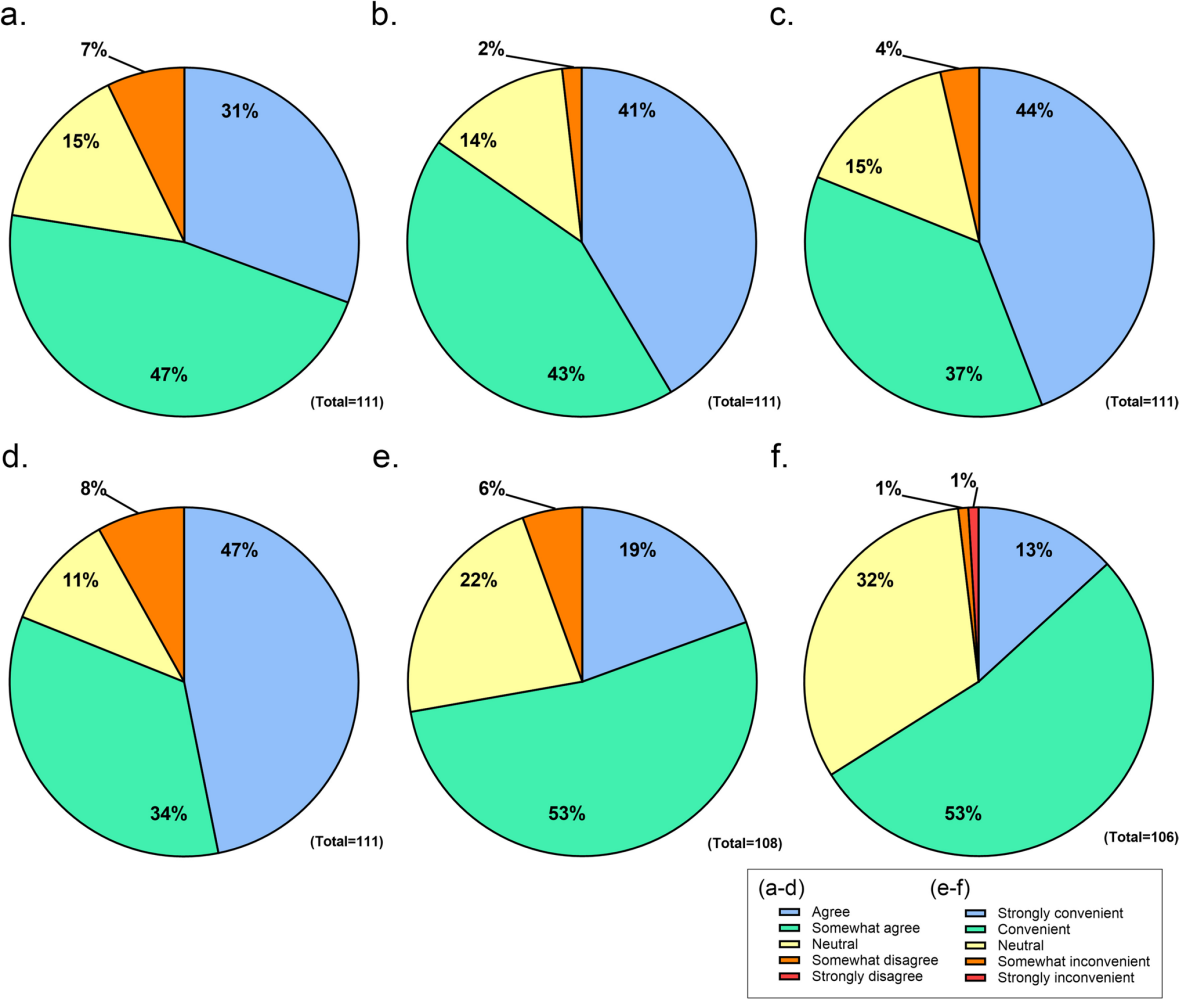
Questionnaire survey on the mHealth application *KaKalink* for participants. Percentages represent levels of agreement or convenience perceptions across different functions

A questionnaire survey using *KaKalink* was administered to all participants at the end of the study. The response rate was 93.3% (111/119), and some items remained unanswered. Regarding the increased knowledge about their medication and BP values, “Agree” was selected by 31% of participants, followed by “Somewhat agree” by 47% and “Neutral” by 15% (Fig. 5a). Regarding becoming more conscious of self-management, “Agree” was selected by 41% of participants, followed by “Somewhat agree” by 43% and “Neutral” by 14% (Fig. 5b). Most participants had few negative responses regarding self-management. Additionally, most participants reported high satisfaction with the use of mHealth application (Fig. 5c–f).

## Discussion

This study revealed that the medication adherence input rate among Japanese patients with CKD was approximately 70%, with higher rates observed in women than in men. Improvements in medication adherence, BP measurements, and BW measurements were anticipated with the use of *KaKalink*; however, no beneficial effects were observed regardless of sex or information reading rate. However, the questionnaire survey revealed that most participants perceived the application to be a useful and highly satisfactory tool for enhancing self-management.

The medication adherence of approximately 70% in our study was consistent with a previous result from a Korean study that reported a similar adherence rate (71.0%) among patients with hypertension [14]. On the other hand, a population-based cohort study conducted in Tsuruoka City, a local area in Japan, reported a higher adherence rate (90.2%) among patients with hypertension [15]. Several plausible explanations for this discrepancy include differences in the participants’ age, sex, and methodologies for calculating adherence rates. In this study, participants were required to tap a button on the application to record their medication intake at the time of administration. This process may have been burdensome for the participants and may have resulted in occasional gaps in the application-based documentation of their adherence to the prescribed regimen. Although their medication adherence “input” rate was evaluated rather than their actual medication adherence rate, the medication adherence input rate may have been lower than the actual medication adherence rate. The lower rates of BP and BW measurements were likely due to similar reasons. The process of recording their BP and BW was somewhat burdensome because they had to manually input their BP or BW values into the application.

The lack of improvement for each rate following the intervention is likely attributable to the excessively long study period and decreasing motivation for input continuity. In this study, the mean age of the participants was 53.3 years, with over 50% of them in their 40 s and 50 s. Younger participants were more likely to use smartphones frequently and be familiar with various applications. Given that participants might have required only a few days to learn how to use the application, the 4-week familiarization period and 8-week non-intervention period (Period A) might have been unnecessary. In a previous study, only 2–8 weeks were sufficient to easily use a smartphone application for older participants with a mean age of 69.5 years. Therefore, it is likely that most participants had already failed to record their medication intake and BP or BW values during Period A because of the prolonged duration before entering Period B. To address the lower input rates using “*Kakalink*” in future studies, the app needs to be improved by various strategies such as the integration of automated data entry, voice-supported data entry, and incentive-based system. These improvements may contribute to enhancing adherence and to increase motivation for input continuity.

The study results demonstrate notable sex differences in medication adherence input rates. During each period, the rates were higher in women than in men. Previous studies and reviews have demonstrated that female sex is an independent predictor of nonadherence to antidiabetic medication, lipid-lowering medication, and evidence-based medication after acute myocardial infarction [16–18]. However, data regarding sex disparities in hypertension, heart failure, and stroke are limited and inconsistent [19–22]. Several biological, treatment-related, psychosocial, socioeconomic, cognitive, and mood-related factors may contribute to sex differences. Another potential reason for this is that women are more likely to experience side effects of drugs. Some studies have investigated the roles of these factors [23]; however, definitive evidence and conclusions are lacking. Nevertheless, the finding that women demonstrated better adherence rates than men was noteworthy. It may be easier for women than for men to monitor their medication use and record measurement data through the application.

Finally, most participants were satisfied with several application functions. In the questionnaire survey, negative responses accounted for less than 10% of each item. In particular, the item “Became more conscious of self-management” received 84% positive responses, which enhanced participants’ interest in health management. Although the app did not contribute to higher input rates, the provision of health-related information to the participants was also highly satisfactory, suggesting that this application can enhance their awareness of health management, at least to some extent. This trend of high satisfaction with the app despite lower usage rates is consistent with previous reports [24, 25].

Our study had some limitations. First, we evaluated the medication adherence input rate rather than the actual adherence rate. Because some participants may have forgotten to record their medication intake in the application, the actual medication adherence input rate in this study could be higher than approximately 70%. Alternative assessment methods, such as pill counts or self-reported questionnaires, should have been considered to validate adherence rates. Second, several factors, such as values of estimated glomerular filtration rate, the presence of diabetes mellitus, the number and type of medications, medication changes during the study period, and timing of medication intake, were not considered in the analysis. Additionally, the participants had different cultural literacy levels and abilities to acquire knowledge from their application. These factors may have affected patient adherence. Although our study primarily focuses on the effects of mHealth application on the medication adherence input rate, we note that future studies with a multicenter approach and larger cohorts should examine responses to the intervention.

In conclusion, the information provided via *KaKalink* did not significantly improve adherence input or changes in BP or BW; however, most participants perceived the application to be a useful and highly satisfactory tool for enhancing their self-management.

## Supplementary Information

Below is the link to the electronic supplementary material.

Supplementary file1 (DOCX 501 KB)

## References

[1] Kalantar-Zadeh K, Jafar TH, Nitsch D, Neuen BL, Perkovic V. Chronic kidney disease. Lancet. 2021;398:786–802. 34175022 10.1016/S0140-6736(21)00519-5

[2] Japanese Society of Nephrology. Essential points from evidence-based clinical practice guideline for chronic kidney disease 2023. Clin Exp Nephrol. 2024;28:473–95. 38713253 10.1007/s10157-024-02497-4 PMC11116248

[3] Imai E, Horio M, Watanabe T, Iseki K, Yamagata K, Hara S, et al. Prevalence of chronic kidney disease in the Japanese general population. Clin Exp Nephrol. 2009;13:621–30. 19513802 10.1007/s10157-009-0199-x

[4] Hanafusa N, Abe M, Joki N, Hoshino J, Kikuchi K, Goto S, et al. Annual dialysis data report, JSDT renal data registry. Renal Replace Ther. 2020;2024:10.

[5] Stewart F, Kistler K, Du Y, Singh RR, Dean BB, Kong SX. Exploring kidney dialysis costs in the United States: a scoping review. J Med Econ. 2024;27:618–25. 38605648 10.1080/13696998.2024.2342210

[6] Nagai K, Iseki C, Iseki K, Kondo M, Asahi K, Saito C, et al. Higher medical costs for CKD patients with a rapid decline in eGFR: a cohort study from the Japanese general population. PLoS ONE. 2019;14: e0216432. 31100069 10.1371/journal.pone.0216432 PMC6524806

[7] Chadban S, Arici M, Power A, Wu MS, Mennini FS, Arango Alvarez JJ, et al. Projecting the economic burden of chronic kidney disease at the patient level (Inside CKD): a microsimulation modelling study. EClinicalMedicine. 2024;72: 102615. 39010976 10.1016/j.eclinm.2024.102615 PMC11247148

[8] Tesfaye WH, Erku D, Mekonnen A, Tefera YG, Castelino R, Sud K, et al. Medication non-adherence in chronic kidney disease: a mixed-methods review and synthesis using the theoretical domains framework and the behavioural change wheel. J Nephrol. 2021;34:1091–125. 33559850 10.1007/s40620-020-00895-x

[9] Nakajima R, Watanabe F, Kamei M. Factors associated with medication non-adherence among patients with lifestyle-related non-communicable diseases. Pharmacy (Basel). 2021;9:90. 33922240 10.3390/pharmacy9020090 PMC8167756

[10] Iuga AO, McGuire MJ. Adherence and health care costs. Risk Manag Healthc Policy. 2014;7:35–44. 24591853 10.2147/RMHP.S19801 PMC3934668

[11] Patrick K, Griswold WG, Raab F, Intille SS. Health and the mobile phone. Am J Prev Med. 2008;35:177–81. 18550322 10.1016/j.amepre.2008.05.001 PMC2527290

[12] Perez-Jover V, Sala-Gonzalez M, Guilabert M, Mira JJ. Mobile apps for increasing treatment adherence: systematic review. J Med Internet Res. 2019;21: e12505. 31215517 10.2196/12505 PMC6604503

[13] Ohya M, Iwashita Y, Kunimoto S, Yamamoto S, Mima T, Negi S, et al. An analysis of medication adherence and patient preference in long-term stable maintenance hemodialysis patients in Japan. Intern Med. 2019;58:2595–603. 31178499 10.2169/internalmedicine.2676-19 PMC6794165

[14] Han E, Sohn HS, Lee JY, Jang S. Health behaviors and medication adherence in elderly patients. Am J Health Promot. 2017;31:278–86. 26730557 10.4278/ajhp.150205-QUAN-709

[15] Matsumoto M, Harada S, Ikuta H, Iida M, Kato S, Sata M, et al. Evaluation of medication adherence among prevalent users in hypertension, dyslipidemia, and diabetes using health insurance claims: a population-based cohort study in Japan. Pharmacoepidemiol Drug Saf. 2024;33: e5855. 39145400 10.1002/pds.5855

[16] Park LG, Ng F, Shim JK, Elnaggar A, Villero O. Perceptions and experiences of using mobile technology for medication adherence among older adults with coronary heart disease: a qualitative study. Digit Health. 2020. 10.1177/2055207620926844. 32489672 10.1177/2055207620926844 PMC7241207

[17] Lauffenburger JC, Robinson JG, Oramasionwu C, Fang G. Racial/Ethnic and gender gaps in the use of and adherence to evidence-based preventive therapies among elderly Medicare Part D beneficiaries after acute myocardial infarction. Circulation. 2014;129:754–63. 24326988 10.1161/CIRCULATIONAHA.113.002658 PMC4351731

[18] Tomida J, Yoshida T, Senda S, Sato T, Nakatsuma A, Iihara N. Statin persistence and adherence among older initiators: a nationwide cohort study using the national health insurance claims database in Japan. Pharmacoepidemiol Drug Saf. 2023;32:873–85. 36960485 10.1002/pds.5622

[19] Erkens JA, Panneman MM, Klungel OH, van den Boom G, Prescott MF, Herings RM. Differences in antihypertensive drug persistence associated with drug class and gender: a PHARMO study. Pharmacoepidemiol Drug Saf. 2005;14:795–803. 16178043 10.1002/pds.1156

[20] Consolazio D, Gattoni ME, Russo AG. Exploring gender differences in medication consumption and mortality in a cohort of hypertensive patients in Northern Italy. BMC Public Health. 2022;22:768. 35428215 10.1186/s12889-022-13052-9 PMC9013154

[21] Kulkarni S, Rao R, Goodman JDH, Connolly K, O’Shaughnessy KM. Nonadherence to antihypertensive medications amongst patients with uncontrolled hypertension: a retrospective study. Medicine (Baltimore). 2021;100: e24654. 33832064 10.1097/MD.0000000000024654 PMC8036043

[22] Venditti V, Bleve E, Morano S, Filardi T. Gender-related factors in medication adherence for metabolic and cardiovascular health. Metabolites. 2023;13:1087. 37887412 10.3390/metabo13101087 PMC10609002

[23] Zucker I, Prendergast BJ. Sex differences in pharmacokinetics predict adverse drug reactions in women. Biol Sex Differ. 2020;11:32. 32503637 10.1186/s13293-020-00308-5 PMC7275616

[24] Lee H, Uhm KE, Cheong IY, Yoo JS, Chung SH, Park YH, et al. Patient satisfaction with mobile health (mHealth) application for exercise intervention in breast cancer survivors. J Med Syst. 2018;42:254. 30402781 10.1007/s10916-018-1096-1

[25] Yu C, Liu C, Du J, Liu H, Zhang H, Zhao Y, et al. Smartphone-based application to improve medication adherence in patients after surgical coronary revascularization. Am Heart J. 2020;228:17–26. 32745732 10.1016/j.ahj.2020.06.019

