# Supplementary material for: Efficacy of a mobile health application on self-management among Japanese patients with chronic kidney disease
Source: Clin Exp Nephrol. 2025 Jun 2;29(10):1354–62. doi: 10.1007/s10157-025-02713-9 (PMC12464019; doi:10.1007/s10157-025-02713-9)
Supplement: Supplementary file 1 — Supplementary file1 (DOCX 501 KB) [file 10157_2025_2713_MOESM1_ESM.docx]

**Efficacy of a mobile health application on self-management among Japanese patients with chronic kidney disease**

Reina Suetsugu-Ishizawa^1^, Hirofumi Sakuma^1^, Motoki Matsuki^1^, Seiji Itano^2^, Hajime Nagasu^2^, Hiroshi Morinaga^3,4^, Haruhito A. Uchida^3,5^, Takashige Kuwabara^6^, Toshiyuki Imasawa^7^, Kazuki Yamada^8^, and Naoki Nakagawa^1^

^1^Division of Cardiology and Nephrology, Department of Internal Medicine, Asahikawa Medical University, Asahikawa, Japan.

^2^Department of Nephrology and Hypertension, Kawasaki Medical School, Okayama, Japan.

^3^Department of Nephrology, Rheumatology, Endocrinology and Metabolism, Okayama University Faculty of Medicine, Dentistry and Pharmaceutical Sciences, Okayama, Japan.

^4^Department of Comprehensive Therapy for Chronic Kidney Disease, Okayama University Faculty of Medicine, Dentistry and Pharmaceutical Sciences, Okayama, Japan.

^5^Department of Chronic Kidney Disease and Cardiovascular Disease, Okayama University Faculty of Medicine, Dentistry and Pharmaceutical Sciences, Okayama, Japan.

^6^Department of Nephrology, Kumamoto University Graduate School of Medical Sciences, Kumamoto, Japan.

^7^Department of Nephrology, National Hospital Organization Chiba-Higashi National Hospital, Chiba, Japan.

^8^Department of Internal Medicine, Kitasaito Hospital, Hokkaido, Japan.

Corresponding author: Naoki Nakagawa, MD, PhD, Division of Cardiology and Nephrology, Department of Internal Medicine, Asahikawa Medical University, Midorigaoka-higashi 2-1-1-1, Asahikawa, Japan.

Phone: +81-166-68-2442, Fax: +81-166-68-2449.

E-mail: [naka-nao@asahikawa-med.ac.jp](mailto:naka-nao@asahikawa-med.ac.jp)

**Supplementary Figure S1.**


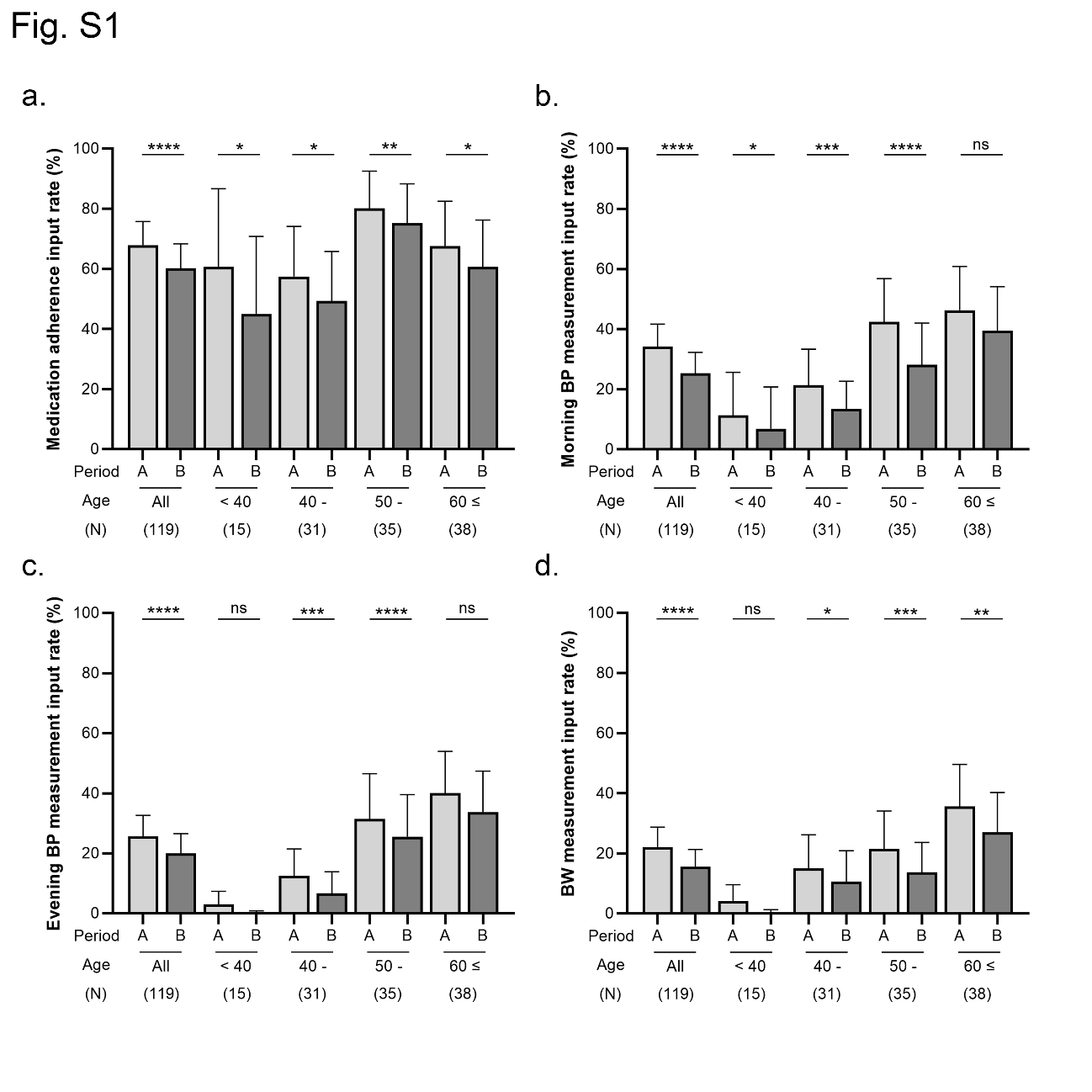


Medication adherence input rates (a), morning BP measurement input rates (b), evening BP measurement input rates (c), and BW measurement rates (d) by subdivided Periods A and B into Periods A1, A2, B1, B2, and B3. Each bar graph and error bar represent the mean and 95% confidence interval. Statistical analysis is performed using analysis of a mixed-effects model analysis and one-way analysis of variance with Holm-Sidak’s multiple comparison test. *p<0.05, **p<0.01, ***p<0.001, ****p<0.0001, compared with Period A1.

**Supplementary Figure S2.**


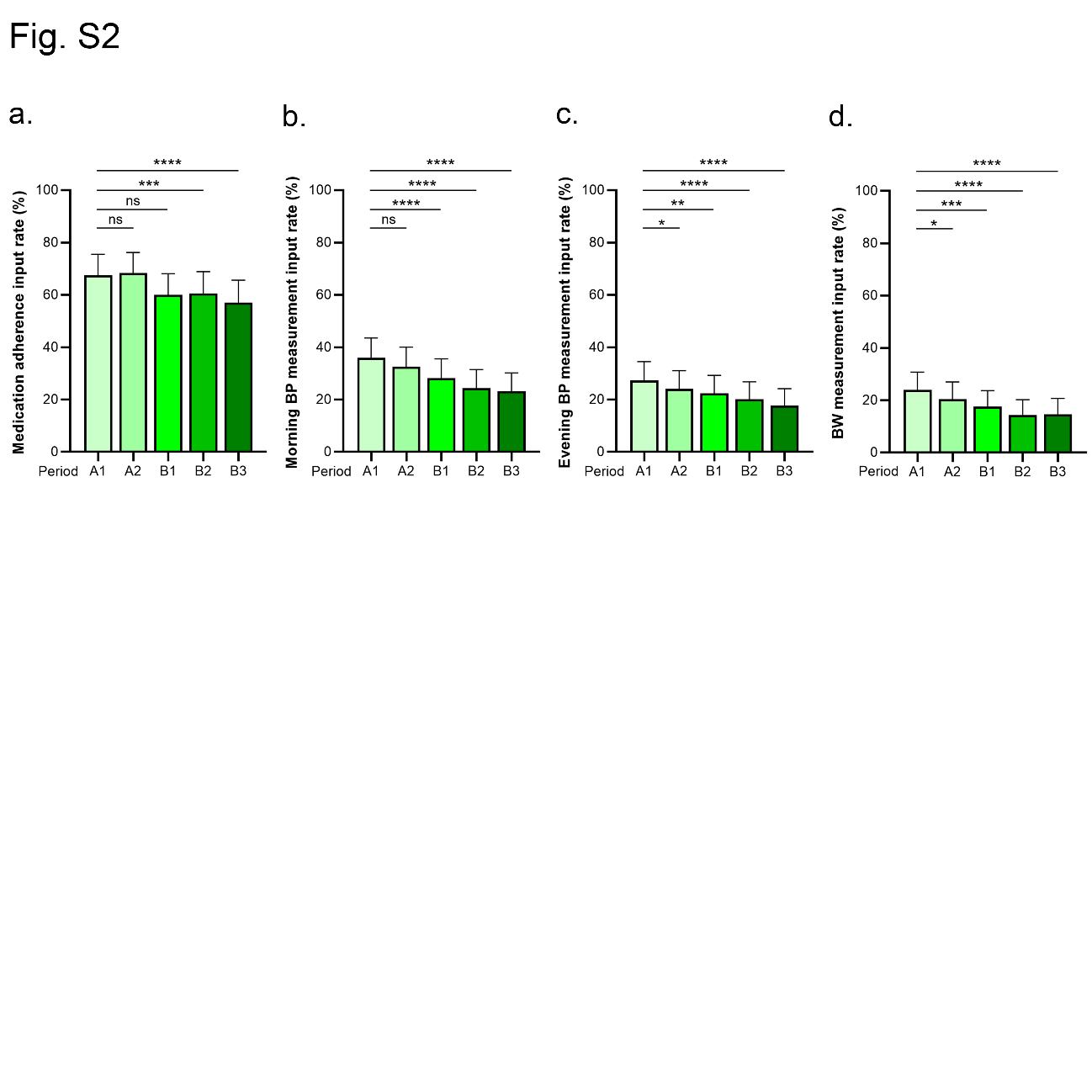


Medication adherence input rates (a), morning BP measurement input rates (b), evening BP measurement input rates (c), and BW measurement rates (d) by subdivided Periods A and B into Periods A1, A2, B1, B2, and B3. Each bar graph and error bar represent the mean and 95% confidence interval. Statistical analysis is performed using analysis of a mixed-effects model analysis with Holm-Sidak’s multiple comparison test. *p<0.05, **p<0.01, ***p<0.001, ****p<0.0001, compared with Period A1.

Supplemental Table 1.

| **Categoty** | **Title** |
| --- | --- |
| **Medication** | What is this medication for? |
|  | What happens if you forget to take madication? |
|  | Are there any side effects of anti-hypertensive drug? |
|  | What happens if you take too mcuh medication? |
|  | Are you following the correct time for taking your medication? |
|  | Is there anything you should not take with anti-hypertensive drug? |
| **Hypertension** | What is the blood pressure? |
|  | Why does the blood pressure elevate? |
|  | The blood pressure varies with seasons. |
|  | Why is the high blood pressure bad for the kidneys? |
| **Chronic kidney disease** | Not only medication, but diet is also important. |
|  | Not only medication, but exercise is also important. |
|  | Be careful for overeating and overdrinking in the holiday season. |
|  | What is the creatinine? |
